# Smart density: a more accurate method of measuring rural residential density for health-related research

**DOI:** 10.1186/1476-072X-9-8

**Published:** 2010-02-12

**Authors:** Peter M Owens, Linda Titus-Ernstoff, Lucinda Gibson, Michael L Beach, Sandy Beauregard, Madeline A Dalton

**Affiliations:** 1 Smart Mobility, Inc., Norwich Vermont 05055, USA; 2 Community Health Research Program, Hood Center for Children and Families, Dartmouth Medical School, One Medical Center Drive, Lebanon, NH 03756, USA; 3 Department of Pediatrics, Dartmouth Medical School, One Medical Center Drive, Lebanon, NH 03756, USA; 4 Department of Community and Family Medicine; Dartmouth Medical School, One Medical Center Drive, Lebanon, NH 03756, USA; 5 Department of Anesthesia, Dartmouth-Hitchcock Medical Center, One Medical Center Drive, Lebanon, NH 03756, USA

## Abstract

**Background:**

Studies involving the built environment have typically relied on US Census data to measure residential density. However, census geographic units are often unsuited to health-related research, especially in rural areas where development is clustered and discontinuous.

**Objective:**

We evaluated the accuracy of both standard census methods and alternative GIS-based methods to measure rural density.

**Methods:**

We compared residential density (units/acre) in 335 Vermont school neighborhoods using conventional census geographic units (tract, block group and block) with two GIS buffer measures: a 1-kilometer (km) circle around the school and a 1-km circle intersected with a 100-meter (m) road-network buffer. The accuracy of each method was validated against the actual residential density for each neighborhood based on the Vermont e911 database, which provides an exact geo-location for all residential structures in the state.

**Results:**

Standard census measures underestimate residential density in rural areas. In addition, the degree of error is inconsistent so even the relative rank of neighborhood densities varies across census measures. Census measures explain only 61% to 66% of the variation in actual residential density. In contrast, GIS buffer measures explain approximately 90% of the variation. Combining a 1-km circle with a road-network buffer provides the closest approximation of actual residential density.

**Conclusion:**

Residential density based on census units can mask clusters of development in rural areas and distort associations between residential density and health-related behaviors and outcomes. GIS-defined buffers, including a 1-km circle and a road-network buffer, can be used in conjunction with census data to obtain a more accurate measure of residential density.

## Background

The built environment has traditionally been the focus of disciplines such as geography, transportation, and city planning. Recently, the potential impact of the built environment on public health has also gained recognition. Of particular interest is whether aspects of the built environment contribute to dietary intake, physical activity, and other behaviors related to obesity, which has increased dramatically in the US in recent decades [1-3]. For example, active travel, the substitution of walking or cycling for motorized transport, is influenced by residential density [4-12], and those living in areas conducive to active travel are at lower risk of being overweight [13-17].

A primary challenge for investigators evaluating the impact of the built environment on active travel and other health-related behaviors is developing valid geographic measures to characterize rural neighborhoods and communities. Studies involving the built environment typically rely on US Census data to measure residential density. Originally developed to collect population data for political purposes, including the specification of US congressional districts, the boundaries established by census geographic units are often unsuited to health-related research.

In large urban areas, continuous development patterns result in relatively consistent census geography. In this context, simple census methods can produce valid measures of residential density that reasonably characterize the built environment at the neighborhood or community scale [1,14,18]. In rural areas, however, development is clustered and discontinuous. Large areas of undeveloped land within rural communities can result in highly irregular units of census geography [19,20]. Because census units are defined by fixed boundaries (roads, streams, etc) rather than land use, the proportion of undeveloped land within individual census units varies widely. For example, the census block boundary of one neighborhood might be a nearby stream, whereas the boundary of a similar neighborhood might extend across thousands of acres of woodland to a stream on the other side of a mountain. Consequently, the use of standard census measures in rural areas can result in vastly different estimates of residential density for two comparably settled communities. Such distortions may potentially affect inferences regarding the impact of the built environmental on health-related behaviors.

The goals of the present study were to (a) evaluate the accuracy of rural residential density as measured by standard census boundaries and (b) determine whether we could develop a GIS-based method using readily available US Census data to more accurately measure residential density in rural areas. We validated the accuracy of the census and GIS-based measures using a data resource containing the exact geo-location of residential structures in the rural state of Vermont. To the best of our knowledge, this information is available only in Vermont, thus providing a unique opportunity to validate alternative methods of assessing rural density.

## Methods

### Overview

We measured residential density in school neighborhoods for all 335 public schools in Vermont using two approaches. The first used conventional census geographic units including tract, block group and block. The second combined census data with two GIS-defined buffers: a 1-kilometer (km) circle around the school and a 100-meter (m) road-network buffer intersecting the circle. The accuracy of each approach was validated against the actual residential density for each school neighborhood based on the Vermont state e911 GIS database, which provides an exact geo-location for all residential structures in the state. Consistent with the data available from Vermont 911 GIS data, all residential densities are expressed in housing units per acre (u/ac) rather than population per area.

### School locations

GIS data downloaded from the Vermont Center for Geographic Information [21] were used to precisely geo-locate all 335 public schools in Vermont within a GIS framework. Manual checks of a random 10% sample were made using orthophotography to verify locational accuracy. The school locations provided the geographic framework for the series of density measurements that follow.

### Measuring residential density using census geography

School neighborhood residential density was calculated using the three smallest standard units of census geography: tract, block group and block. Each school location was matched to a specific census tract, block group and block using the intersect tool in ArcGIS 9.2 (ESRI, Redlands CA). Residential density was calculated for each school neighborhood by dividing the number of housing units by the number of land area acres within each of the three census geographic units (u/ac). US 2000 Census data and related geographic (TIGER) files were downloaded from the ESRI website [22].

### Measuring residential density using GIS buffers

We used two GIS buffers to refine residential density calculations. First, a 1-km radius circle buffer (approximately 776 acres) centered at the school site was used delineate the school neighborhood. We used the intersect tool of ArcGIS to select census blocks that were wholly or partially contained within the 1-km circle buffer. When a census block fell entirely within the buffer, 100% of the housing units were counted. When a census block was only partly contained within the buffer, the housing unit counts were pro-rated according to the percentage of the census block area contained within the buffer. Residential density was calculated as the number of housing units divided by the number of acres within the buffer (u/ac).

Secondly, using the buffer tool of ArcGIS, a 100-m road-network buffer was used to factor-out wetlands, forests, farmland and other undeveloped areas. This buffer was based on field and map analyses that showed nearly all residential development is located within a 100-m (325 feet) zone of the road network. The road-network buffer delineated areas within 100 meters of the centerline of named roads; areas adjacent to limited access roads and unnamed roads were excluded. This road-network buffer was then intersected with the 1-km circle buffer around the school using the intersect tool in ArcGIS. Residential density was calculated by dividing the number of housing units by the number of developed acres within the buffer (u/ac).

When a school neighborhood overlapped a state boundary (*n *= *4*), both the 1-km circle buffer and 100-m road-network buffer included only the census data and geographic area contained within Vermont, preserving the ability to validate the measure using Vermont e911 data.

### Measuring residential density using e911 structure data

We used Vermont's e911 data to calculate actual residential density (u/ac) based on the exact geo-location of residential structures and developed land area in each school neighborhood. As with the GIS-refined measure, developed land was defined as area within 100 meters of the centerline of named roads and the school neighborhood was delineated with a 1-km circle radius buffer. Using the ArcGIS intersect function, the 1-km circle buffer layer was intersected with the residential structure layer to derive an exact count of residential structures in each school neighborhood. The e911 data distinguish nonresidential structures from residential structures and distinguish types of residential structures (single family, multi-family, mobile home, etc). Our field observations indicated that most of the multi-family structures were small residential buildings containing on average 2 to 3 residential units. Thus, we applied a coefficient of 2.5 to estimate housing unit counts in multi-family structures. Multi-family structures accounted for less than 10% of total structures in the majority (N = 318) of school neighborhoods. More than 60% of the multi-family structures in the sample were located in the Burlington and Rutland school neighborhoods (N = 17), where they accounted for over 40% of the structures. Residential density for each school neighborhood was calculated as u/ac.

### Validation of residential density measures

To assess the correspondence between each method and actual residential density, we plotted the log of e911 residential density against the log of each measure described above (census tract, census block group, census block, 1-km circle buffer, 1-km circle intersecting a road-network buffer). In each plot, we include the identity line, which has an intercept of 0, a slope equal to 1, and represents an exact agreement between the two measures. We also used linear regression to regress the log of e911 residential density on the log of residential density from each method. The log scale was chosen because it provided a more constant error structure across the range of residential densities with the values more uniformly spread. The R-square, mean square error (MSE), slope, and intercept are reported for each of the five regression models. The MSE is the variance estimate for the difference between e911 residential density data and the independent variable; it reflects both model error and lack of fit from the identity line such that lower values indicate higher agreement between the two variables. 95% confidence intervals for the intercept and slope values are reported to indicate whether they were significantly different from 0 or 1, respectively.

### Qualitative study

We created comparative maps of the residential settlement patterns for each of six selected school neighborhoods. For each neighborhood, e911 residential density values and the comparative maps were verified using orthophotography, field data, and GIS mapping of actual residential structures. The six neighborhoods show the disparity of census measures across the spectrum of residential density and settlement patterns. The examples were chosen to illustrate three pairs of neighborhoods in which census data suggest identical residential density, but actual e911 data indicate divergent densities. Manual checking of each of the six school neighborhoods confirmed the accuracy of e911 residential densities. Aerial orthophotography ([23] viewed at 1000 feet/200 meter scale window) confirmed the concurrence of GIS maps with "on-the ground" development patterns.

## Results

**Figure 1:**
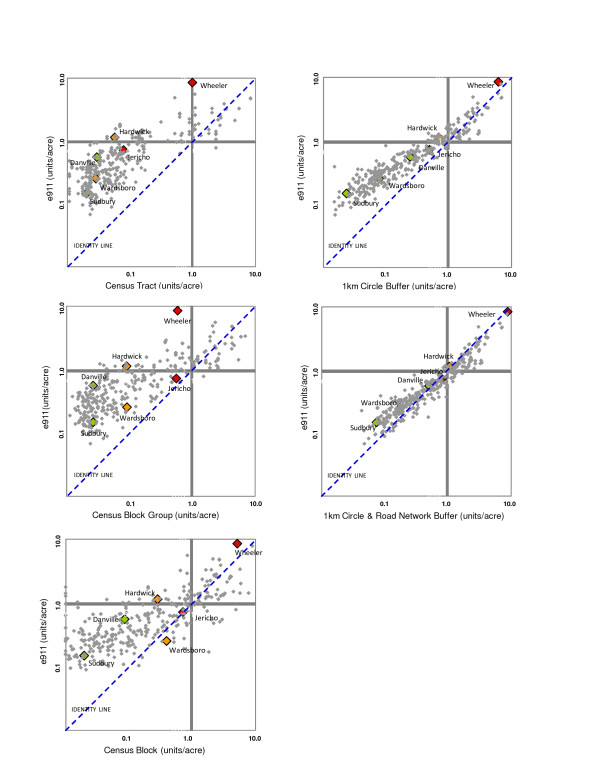
**Residential density from e911 data plotted against residential density derived from census and GIS-buffer measures**. E911 density values are based on actual geo-location of all residential structures in the state of Vermont. Both the x and y axis are on the logarithmic scale but actual density values are displayed. The identity line has an intercept of 0, a slope equal to 1, and represents an exact agreement between the two measures. Six highlighted school neighborhood sites are illustrative pairs in which residential densities based on census block group are comparable, but widely divergent when based on other measures.

Figure 1 plots the log of e911 residential density versus census and GIS measures for the 335 school neighborhoods. In general, calculations based on census geographic units underestimate residential density in more rural areas (e911 residential density of less than 1 u/ac). This is most clearly illustrated in the first panel of Figure 1, where all but one of the rural residential densities based on census tract are to the left of the identity line. In addition, the degree of variation around the identity line is not consistent across the range of residential densities, such that the variability is highest in rural areas. Of the three census geographic units, the block group has the highest agreement with e911 residential density. However, even with this measure, only 51 of 335 sites had residential densities within 50% of the e911 values. Using the census block as the geographic unit, 12 school neighborhoods were determined to have residential densities of 0 because there were no housing units within the block. In contrast, the e911 residential densities of these neighborhoods ranged from 0.2 to 1.8 u/ac.

Residential density measures based on GIS methods are more tightly distributed around the identity line than those based on census units (Figure 1). The pattern of underestimating residential density in rural areas remains apparent for the 1-km circle buffer calculation but shows improvement over the census calculations. As illustrated in Figure 1, the best agreement with e911 residential density was obtained when the 1-km circle buffer was intersected with the road-network buffer. For this measure, residential density is overestimated in the higher residential density areas (> 1 u/ac), but the extent to which this occurs is reduced compared to the census measures.

Table 1 provides the statistics from the individual regression models used to predict e911 residential density values from each of the residential density measures. The R-square values for the census measures range from 0.61 to 0.66, indicating that residential densities based on census geography alone explain between 61 and 66% of the variation in actual residential density. In contrast, the GIS buffer calculations explain approximately 90% of the variation. The MSE, which reflects both the model error and lack of fit with the identity line, is substantially lower for both of the GIS measures compared to the three census measures, indicating that the GIS-refined measures more closely correspond to e911 residential density. The slope is closer to 1 for the 1-km circle and road-network buffer, which suggests that this measure might perform slightly better at the extremes of residential density than the 1-km circle buffer. The intercept is also closer to 0 for the 1-km and road-network buffer than for the 1-km circle buffer, indicating that the former also would perform better when the residential densities are closer to 1 u/ac. However, this difference is not stark. Overall, the calculation based on the 1-km circle and road-network buffers provides the closest approximation of actual residential density determined by e911 data.

**Table 1:** Evaluation of agreement between residential density calculations and actual residential density determined by e911 data

Residential Density Calculation	R^**-**^squared^**#**^	**MSE**^**@**^	**Slope**^**#**^	(95% CI)	**Intercept**^**#**^	(95% CI)
*Census Geography*						

Census tract	0.63	0.32	0.49	(0.45, 0.53)	0.59	(0.46, 0.71)

Census block group	0.66	0.29	0.44	(0.41, 0.48)	0.36	(0.26, 0.46)

Census block	0.61	0.34	0.41	(0.38, 0.45)	0.04	(-0.05, 0.13)

*GIS-refined Measure*						

1-km circle buffer	0.89	0.09	0.59	(0.57, 0.61)	0.21	(0.17, 0.26)

1-km circle and road network buffer	0.90	0.08	0.75	(0.73, 0.78)	-0.11	(-0.11, -0.07)

^#^R-squared, slope, and intercept are from models in which both the calculated residential density and e911 residential density are transformed to the log scale.

^@^MSE is the variance estimate for the difference between calculated residential densities and e911 residential density; it reflects both model error and lack of fit from the identity line as illustrated in Figure 1.

We selected a subset of six school neighborhoods to illustrate the disparity between residential density determined by census measures versus e911 data. The six school neighborhoods are highlighted in the scatter plots shown in Figures 1. The correct relative rank of residential density (i.e., highest to lowest) as determined by e911 data is preserved in the calculations based on GIS buffer methods. In contrast, because the calculations based on census measures introduce inconsistent error, the relative ranking of residential densities can be distorted (Figure 1).

**Figure 2:**
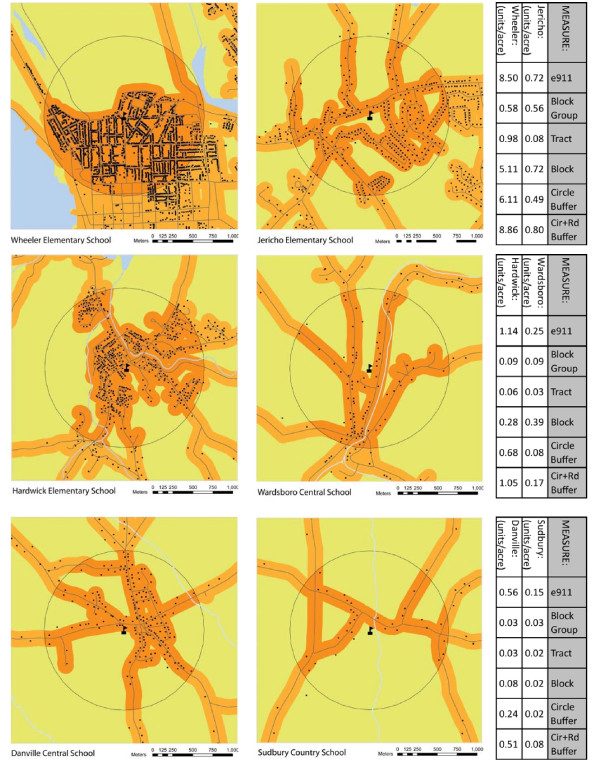
**Comparative maps and residential densities of three school neighborhood pairs illustrating differences in density calculations**. The three school neighborhood pairs were chosen to illustrate scenarios in which residential densities appear to be comparable based on census block group but are substantially different based on e911 data.

In Figure 2, the residential settlement patterns of the six school neighborhoods are arrayed in three pairs, representing the high, middle and low distribution of residential density. The residential density within each pair of neighborhoods is nearly identical when measured by census block group but divergent based on e911 data. These maps visually illustrate the disparity in residential settlement patterns that may not be captured when using census geographic units to calculate residential density. For instance, Wheeler and Jericho school neighborhoods have similar block group density values of .58 u/ac and .56 u/ac, respectively, but the actual densities based on e911 data are 8.50 u/ac and 0.72 u/ac, reflecting a 10-fold difference in the density of the two communities. This difference in density is illustrated in the maps by the denser pattern of dots (i.e. residential structures) in Wheeler compared to Jericho. The other census measures (tract, block) also show an inconsistent relative agreement with the e911 values.

## Discussion

We used Vermont statewide e911 data to assess the accuracy of census and GIS-refined residential density calculations of school neighborhoods in predominantly rural areas. Our findings indicate that conventional census methods substantially underestimate residential density in the rural environment. The underestimation is particularly acute where residential density is less than 1 u/a, which generally corresponds to areas more rural in character. In addition, because the error is inconsistent across geographic areas, even the relative ranking of residential density may be incorrect.

The census was established by the US constitution to measure population distribution for the purpose of apportioning congressional districts and, subsequently, federal funds [24]. As such, census geography is not necessarily well specified for other applications, such as public health research [3,25]. Nonetheless, census data are often used for health-related research because they are readily available and there is no charge for their use. Because of the relative consistency of census geography in urban areas, census data may accurately characterize neighborhoods and small geographic areas in metropolitan settings. However, in discontinuously settled rural areas, even the smallest census geographic units typically mix developed areas with substantial areas of undeveloped land, such as woodlands, wetlands, and other uninhabited areas [20]. Potentially compounding the problem, census units also vary considerably by size; e.g., from 5 to 25,000 acres within a single VT county. Consequently, in rural areas, the proportion of developed land within a census unit can vary substantially. Estimates of residential density, typically calculated by averaging the number of housing units over the acreage of the census unit, can mask clusters of development [25]. Such errors of measurement can distort associations between residential density and health-related behaviors or outcomes. For example, underestimating the size or density of the source population could spuriously inflate the estimated proportion of people affected by an exposure or outcome of interest. Additionally, underestimates of residential density in rural areas could result in inadequate allocations of health-related services.

The inaccurate census-based calculations of rural residential density shown in our study are consistent with the modifiable areal unit problem (MAUP) [26], the bias arising when aggregated data are based on geographic units of varying sizes. In general, MAUP demonstrates that smaller areal units will reveal more varied but less generalizable findings, whereas larger areal units will create more homogeneous findings that mask local differences. While the error can be minimized by the individual-level study of neighborhoods [27], such data are not available for most rural areas and onsite assessments are not always feasible. The results of our study provide a method of avoiding this bias through GIS tools that apply uniform areal units of a scale appropriate to the subject of study.

The 1-km radius circle buffer used in this study is a reasonable neighborhood scale [19] with an accessible pedestrian domain (10-15 minute walking time from center to perimeter). While a network buffer distance can be useful in urban settings, we chose a straight-line radius because it does not require sophisticated software and our preliminary work showed little difference between the network and straight-line buffers when applied to rural areas. The 1-km circle buffer showed strong agreement with the e911 residential densities in urbanized communities and produced correct relative rankings of density in rural communities. However, residential density was underestimated due to the inclusion of undeveloped land. Intersecting the circle with a road-network buffer removed much of the undeveloped land and improved agreement with e911 residential density.

The availability of e911 data in Vermont provided a unique opportunity to evaluate the accuracy of various methods of calculating rural residential density. However, e911 data do not specify the number of units in multi-family structures. This is not a problem in rural areas with few multi-family structures, but could introduce error where such structures are more common. Based on field observations, we used a coefficient of 2.5 to estimate the average number of units in multi-family structures. This reflects the fact that multi-family structures in rural areas with older housing stock, such as those included in our study, tend to be small residential buildings rather than apartment complexes. However, newer residential development, especially those closer to urban areas, might include structures with a higher number of units, underscoring the importance of field observation to "ground truth" research assumptions. It is important to note that although we measured residential density in housing units per acre, our methods are equally appropriate for any other measure of density that can be derived from US census data (e.g. population per area).

In summary, our findings demonstrate that using standard census units to calculate residential density both underestimates and inaccurately ranks relative residential densities in rural areas. GIS techniques that incorporate a simple radius circle and road-network buffer provide excellent agreement with residential densities based on exact geo-location data for residential structures. A major advantage using GIS buffers is that they allow researchers to adjust the geographical frame of reference to match the unit of interest. Thus, this method would be valuable to any rural research application using census data where geographic scale matters.

## Abbreviations and Definitions

GIS: Geographic Information System.

## Competing interests

The authors declare that they have no competing interests.

## Authors' contributions

As corresponding author (MD), I had full access to the data and final responsibility for submitting the manuscript. Author contributions are as follows: PO, LTE, LG and MD participated in the conceptualization and design of the study, data analysis, interpretation of the findings, and manuscript preparation; MB participated in the conceptualization and design of the study, data analysis, and interpretation of the findings. SB participated in the data analysis and interpretation of the findings. All authors have seen and approved the final version.
